# 5-Hydroxymethylfurfural (HMF) Production from Real Biomasses

**DOI:** 10.3390/molecules23092201

**Published:** 2018-08-31

**Authors:** Federica Menegazzo, Elena Ghedini, Michela Signoretto

**Affiliations:** CATMAT Lab, Department of Molecular Sciences and Nanosystems, Ca’ Foscari University Venice and Consortium INSTM, RU of Venice, Via Torino 155, 30172 Venezia, Italy; fmenegaz@unive.it (F.M.); gelena@unive.it (E.G.)

**Keywords:** direct HMF production, raw biomass, biomass feedstock, biorefinery, edible biomass, lignocellulosic biomass, food wastes

## Abstract

The present paper reviews recent advances on the direct synthesis of 5-hydroxymethylfurfural (HMF) from different kinds of raw biomasses. In particular, in the paper HMF production from: (i) edible biomasses; (ii) non-edible lignocellulosic biomasses; (iii) food wastes (FW) have been reviewed. The different processes and catalytic systems have been reviewed and their merits, demerits and requirements for commercialisation outlined.

## 1. Introduction

How to provide the chemical industry with new tools to transform biomass into chemicals in a 360-degree sustainable way is a challenge for modern scientists [1]. In fact, with diminishing fossil resources, the development of new technologies to exploit versatile and renewable biomasses as alternative feedstock for platform chemicals has received more attention than ever. Biomasses can be considered a renewable resource because they can be replenished over a relatively short timescale and they are essentially limitless in supply [2]. In the general framework, the upgrading and valorisation of the C6 biomass fraction represents a specific relevant issue. Indeed, hexoses are the most abundant monosaccharide existing in Nature. Nowadays, the catalytic transformation of hexoses into furans is very interesting and the furanic products available with this strategy include 5-hydroxymethylfurfural (HMF), 2,5-diformylfuran (DFF), 2,5-furandicarboxylic acid (FDCA), 2,5-*bis*(hydroxymethyl)furan (BHF), and 2,5-dimethylfuran (DMF) [3]. These can be used as starting materials for new products as well as for the replacement of oil-derived chemicals.

**Figure 1:**
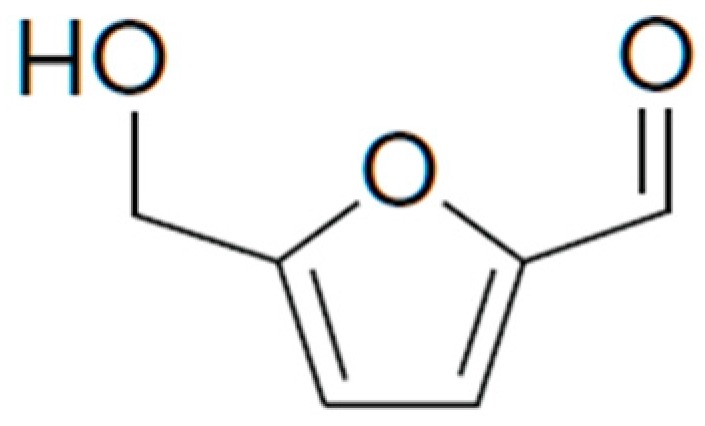
5-Hydroxymethylfurfural (HMF) structure.

HMF (Figure 1) is a multifunctional molecule because it is at the same time an aromatic aldehyde, an aromatic alcohol and a furan ring system. HMF is a versatile intermediate that can be further transformed into a high value-added chemicals. For these reasons it has been called a “sleeping giant” in the field of intermediate chemicals from bio-based feedstocks [4]. Unfortunately, HMF is not stable for long periods and therefore it cannot be stored for extended periods. Furthermore, a high HMF purity is essential even for storage over short time periods because even small impurities promote rapid aging due to dimer and oligomer formation [5].

**Figure 2:**
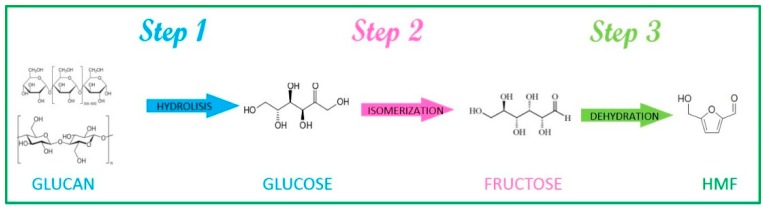
Schemes of the reaction steps from glucan to HMF.

In principle HMF production seems easy—it is synthesized by the loss of three molecules of water from a hexose material in an acid catalyzed reaction. However, in fact the synthesis of HMF synthesis is quite complicated because, actually, many other reactions occur. On paper carbohydrates can be converted to HMF through a three-step reaction as schematized in Figure 2:

Step 1: hydrolysis of glucan (a glucose-based polymer, e.g., cellulose and starch) to glucose catalyzed by a Brønsted acid;
Step 2: isomerisation of glucose to fructose mediated by a Lewis acid;
Step 3: dehydration of fructose to HMF facilitated by a Brønsted acid.

In addition, side reactions that reduce the HMF yield occur, including rehydration of HMF to form levulinic and formic acids, and cross-polymerization. Of course, the key to achieve high HMF yields is to promote the desirable reactions and suppress the others by choosing suitable process conditions and the proper catalytic system.

Many scientific investigations have demonstrated that HMF can be obtained not only from fructose, sucrose and inuline [6,7,8,9,10,11,12,13,14,15,16,17], but also from glucose via isomerisation to fructose [3,8,18,19,20,21], from polysaccharides (inulin, starch, or cellulose) [3,22,23,24,25,26,27] and directly from real biomasses too. Ideally, the best process is in fact a one-pot transformation from a real biomass to HMF. The present paper reviews recent advances on the direct synthesis of HMF from different kinds of raw biomasses.

The direct use of real biomass can indeed lead to less energy consumption and less CO_2_ emissions. In practice, using a raw plant biomass avoid the pretreatment steps that are required in preparing the purified bio-based feedstocks. However, for scientists the conversion of biomass is much more challenging than model carbohydrates because the decomposition behavior of the feedstock depends on the interactions between the cellulose, hemicellulose, and lignin. Up to now, most studies have still focused on using feedstocks exhibiting simple and regular molecular structures, due to their easier catalytic mechanisms. However, from the viewpoint of practical applications, direct conversion of real biomass is highly desirable because they are the ideal feedstock to be used for any commercial unit to economically produce bio-based products. The first studies regarded edible biomasses, but for these there are very strong socio and economic issues and therefore the use of edible feedstocks appears unsustainable. The upgrading of lignocellulosic biomass wastes (e.g., wood, straw, energy crops, etc.) is a good opportunity because they avoid competition with the food sector and usually do not require as much as land to grow [28]. On the other hand, food wastes represent a massive annual dissipation of resources and incurs a high carbon footprint, as every year, the food chain results in large amounts of wastes and their valorization attracts great attention. The generation of commercial and industrial food wastes shows a growing trend globally. About 45–55% of the municipal solid wastes is due to food wastes [29]. As the market price of HMF is highly sensitive to the feedstock cost, replacing fructose by a waste-derived alternative could improve the economics of the process.

**Figure 3:**
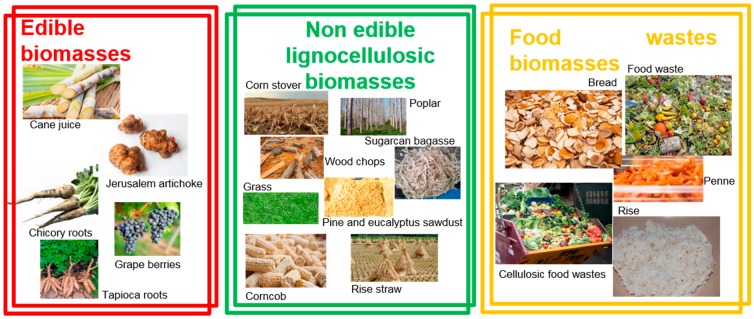
Schematization of the three sections of the review.

Current commercial production of HMF predominantly relies on the syrups extracted from energy crops [30]. The AVA Biochem HTC process can produce HMF at various levels of purity—up to 99.9%. However, use of different biomass resources or food wastes as an alternative feedstock could enable more sustainable manufacturing practices. We have focused our review on the use of raw biomass. In particular, as schematized in Figure 3, in this paper we have reviewed HMF production from:

(i) Edible biomasses;
(ii) Non-edible lignocellulosic biomasses;
(iii) Food wastes (FW).

The different processes that have been developed for HMF production from different feedstocks have been reviewed and their merits, demerits and requirements for commercialization outlined.

## 2. HMF Production from Edible Biomasses

A pioneering patent of 1948 regards HMF synthesis from crude cane juice [31], which contains mainly hexoses and disaccharides (62 wt% of biomass). The inventors used water as a solvent and oxalic or phosphoric acid as a catalyst. Yields of 11 and 9 wt% were obtained, respectively, after 1 h of reaction at a temperature between 140–70 °C. Later, in 1969, another patent reported HMF synthesis from the same cane juice but also on bagasse and hydrolyzed wood waste [32]. Reactions were performed at 270 °C using an acid catalyst. Best results were obtained with 3% Al_2_(SO_4_)_3_·18H_2_O based on solids plus 4.6% concentrated H_2_SO_4_. Using a contact time of 8 s, HMF yields of up to 44% can be obtained.

Jerusalem artichoke (*Helianthus tuberosus*) is a biomass very rich in polyfructans that can be directly transformed into HMF. It has been reported on 1983 [33] that the freshly harvested tubers were washed and grated, and then polyfructans were extracted using hot water containing sulphur dioxide (0.1%). The selective synthesis of HMF was carried out in a triphasic system using a water-ion-exchanger-extracting solvent. In the case of polyholosides, particularly inulin, the ion exchange resin catalyzes both the hydrolysis of the natural plant polymer and the dehydration of the monomer to HMF. Using water as solvent and a strongly acidic macroporous resin with a polystyrene matrix (Lewatit SPC 108; 1.3 m_eq_. H^+^/mol inulin), a HMF yield of 57 wt% was obtained after 15 h of reaction.

Using the same Jerusalem artichoke tubers [34], a more recent study proposed a methodology for HMF production based on the Fenton reaction. In such oxidative process, when Fe^2+^/Fe^3+^ co-exists in a mixture of 2-butanol and water, hydrogen peroxide is converted into a hydroxyl radical, which is one of the most powerful oxidants, according to the reaction:

H_2_O_2_ + Fe^2+^→Fe^3+^ + OH + OH^−^

As regards pre-treatment, Jerusalem artichokes were simply crushed, added in the reactor with FeCl_2_/H_2_O_2_ (pH 3.0–3.5) and heated. The optimum temperature was found to be 180 °C, which resulted in the maximum yield (35%). In the same study, the optimal reaction conditions were identified by using a statistical approach. In particular, the experimental factors were optimized with the Box-Behnken Design (BBD) by the Response Surface Methodology (RSM). Three parameters, that is: (i) reaction time, (ii) Fe^2+^ concentration, (iii) hydrogen peroxide amount, were selected as experimental factors, and HMF yield as the response parameter. A maximum predicted yield of 46% was obtained under the optimal conditions, i.e., after 90 min of reaction at 180 °C with a 0.47 M H_2_O_2_ and 1.3 mM Fe^2+^ concentrations.

Also raw chicory roots can be used for their direct transformation into HMF because they are very rich in inulin (70–75% by dry weight), which is a mixture of linear fructose polymers and other oligosaccharides, linked by β-2,1-glycosidic bonds. In particular, about 80–90% of the total carbohydrate contents in chicory root are comprised of inulin-type fructan. These components can be used for HMF synthesis because their hydrolysates are mainly fructose units that are easily convertible to HMF. 

A 1988 patent [35] claimed that it is possible, using water as solvent, to prepare HMF in great purity from chicory roots. In order to pretreat it, the biomass was ground and then mixed with water and sulphuric acid (pH 1.8). The mixture was heated at 140 °C in a stirrer autoclave for 2 h of reaction. In this way, the yield in HMF is 9%.

**Figure 4:**
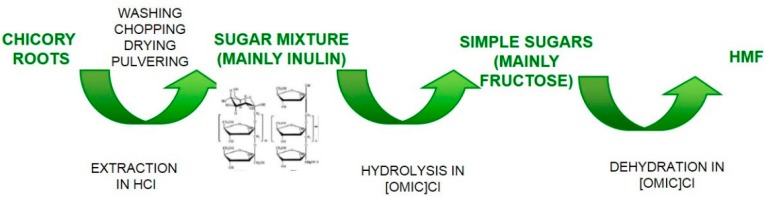
Scheme of the direct process from chicory roots to HMF [36].

The same chicory roots were employed in 2011 in a study for the direct HMF synthesis using HCl and the ionic liquid (ILs) 1-octyl-3-methylimidazolium chloride ([OMIM]Cl), as schematized in Figure 4 [36]. As regard as the pretreatment of chicory roots, they were washed, chopped, dried, pulverized and finally treated with HCl in order to hydrolyze inulin. HCl played an important role in this step, that is for the hydrolysis of chicory sugar polymers. In fact, without HCl there is no inulin conversion, while best results were obtained with 0.3 M HCl (conversion of about 60 dwt%). The highest HMF yield (around 51 dwt%) was obtained in a reaction mixture containing 50% of the reaction solvent ([OMIM]Cl + ethyl acetate) and 50% of the extract prepared in 0.3 M HCl solution by undergoing the reaction at 120 °C for 1 h. Again, higher HCl concentration lead to lower HMF yield. The addition of CrCl_2_ catalyst decreased HMF yields. This suggests that the action of CrCl_2_ on the HMF production depends on carbohydrate type and that probably there is an inhibitory action by CrCl_2_ on the hydrolysis of chicory inulin polysaccharides and/or other unknown chemical reactions involved in sugar dehydration. The highest productivity of HMF was achieved using a chicory root extract prepared from 20% (*w*/*v*) root biomass concentration. The authors have noted that higher root concentrations (such as 30% and 40%(*w*/*v*)) showed highly viscous property in the acidic solution and thus are difficult to handle. 

**Figure 5:**
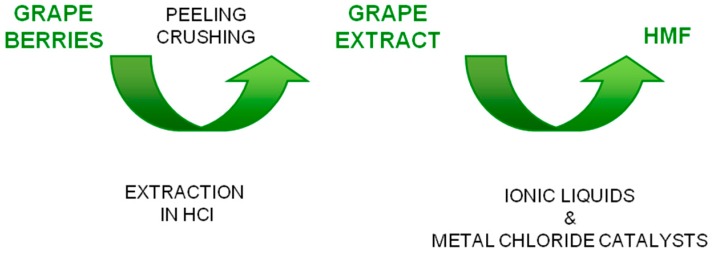
Scheme of the process from grape berries to HMF [37].

It has been reported that also crop plants containing a high amount of soluble sugars, such as grapes, can be a good source of raw biomass materials for HMF production [37]. In fact, the high amounts of soluble sugars which are present in grapes, are more quickly converted to HMF than other plant biomass. Using grape berry biomass as a raw biomaterial, a two-step HMF synthetic process was developed using IL solvents with metal chloride catalysts, as schematized in Figure 5. As regard as pretreatment, grape berries were peeled and crushed. Then the slurry was squeezed, centrifuged and stored at −80 °C. Over 90% of total soluble sugars was found to be fructose and glucose. The authors investigated the effects of:

(i) IL solvents (1-methyl-3-octylimidazolium chloride ([OMIM]Cl), 1-hexyl-3-methylimidazolium chloride ([HMIM]Cl), 1-ethyl-3-methylimidazolium chloride ([EMIM]Cl);
(ii) reaction temperatures (80, 100 and 120 °C);
(iii) chloride catalysts (CrCl_2_, CoCl2·6H_2_O, NiCl2·6H_2_O, ZnCl_2_, MgCl_2_);
(iv) HCl concentrations (0, 0.1, 0.3, 0.5, and 1 M).

They found that the addition of the ILs solvents to the grape biomass extract led to a higher HMF yield, but the degree of its yield enhancement depended on HCl concentration and reaction temperature. [OMIM]Cl was the best solvent. Addition of HCl or metal chlorides alone showed little improvement. The positive effect of HCl has been attributed to a combined role of HCl as a Brønsted catalyst and the dual property as cation and anion of ILs. The highest HMF yield (about 100 mg HMF/mL of grape biomass extract) was obtained using 0.3 M HCl, [OMIM]Cl, and CrCl_2_ at 100 °C for 3 h [37]. Grape berries are an interesting biomass because their sugars, mainly fructose and glucose, are quickly converted to HMF without a depolymerization process. The high yields of HMF from the grape berries is due both to the high amounts of soluble sugars and to the weak coordination of the ILs with the sugars, which facilitates the rapid formation of a complex between the sugars and metal chlorides.

**Figure 6:**
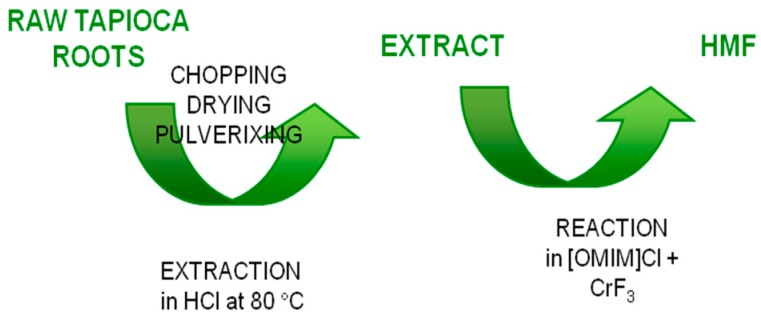
Scheme of the process from raw tapioca roots to HMF [38].

The same authors used the IL 1-octyl-3-methylimidazolium chloride and CrF_3_ catalyst for the HMF synthesis from raw tapioca roots. The latter are an interesting biomass for HMF production because they are very rich in starch (about 70–75% by dry weight). Moreover, tapioca plant is an annual shrub growing in tropical and subtropical regions, and no systematic farming is required [38]. A two-step process (Figure 6) was investigated. The authors studied the hydrolysis efficiency by monitoring the physicochemical changes of biomass components using Fourier transform-infrared spectroscopy (FT-IR) and scanning electron microscopy (SEM). The FTIR absorbance bands confirmed the presence of starch and other soluble sugars in the acidic biomass extract. SEM images of the tapioca powder sample shows globular starch granules with irregular shapes (5–15 μm) and biomass debris. SEM image showed that the efficiency of the biomass hydrolysis is time-dependent. When the extraction time elapsed, the number of starch granules gradually decreased and finally were destroyed, implying that the destruction degree of the starch granules may depend on the reaction time. The concentration of CrF_3_ significantly affected the performances. A HMF yield of about 53 dwt% was obtained using 1% CrF_3_. The combined effect of CrF_3_ and other chromium halides, such as CrBr_3_, CrCl_2_, and CrCl_3_, resulted in higher yield than CrF_3_ alone. Best results were obtained for the addition of CrBr_3_ to CrF_3_, probably due to a synergistic effect.

Therefore, as schematized in Table 1, different edible biomasses have been investigated in the past for HMF production, some with good results. Mainly homogeneous catalysts, recently associated with ILs, have been investigated. However, when considering the feasibility of the HMF production, it is important to take into account the environmental/energy costs associated with the process, such as for example the cost of the biomass feedstock and the potential for increased HMF production. All the socio and economic issues connected to edible biomass are very strong, first of all the use of arable land for biomass production, because it is in competition with the food sector. Therefore, the use of edible feedstocks appears unsustainable. This is confirmed also by the scaricity of scientific works in the last few years about edible feedstocks.

## 3. HMF Production from Non-Edible Lignocellulosic Biomasses 

Both commercial and sustainable issues dictate that efforts should focus on HMF production from lignocellulosic biomass rather than from edible products [39]. Lignocellulosics are a major type of biomass consisting mainly of cellulose (38–50%), hemicellulose (23–32%) and lignin (15–25%) [40]. Agricultural residues, energy crops, forestry residues and so on, are all different types of lignocellulosic biomass, but the exact composition varies depending on the source. Despite the potential of lignocellulosic feedstocks, the challenges their use impose have made it not convenient from both efficiency and economic point of view until now. Only in the last ten years, have some studies appeared about direct transformation of lignocellulosic biomass into HMF (Table 2). 

### 3.1. Liquid Ionic as a Solvent

**Figure 7:**
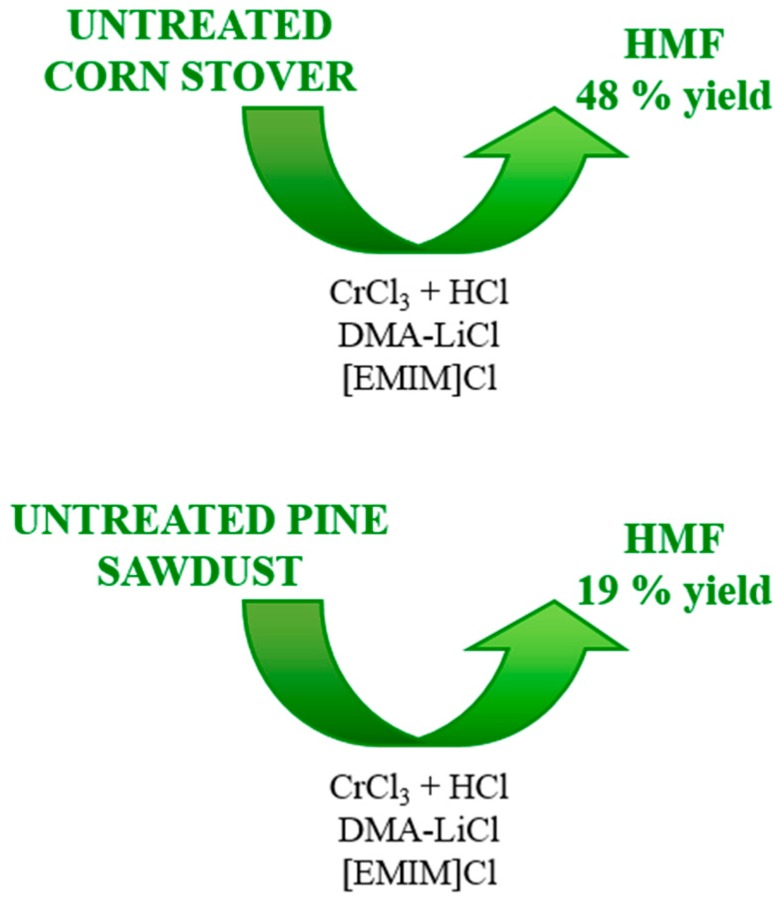
Schemes of the process from untreated corn stover or pine sawdust to HMF [19].

In 2009 corn stover and pine sawdust [19] were transformed into HMF by a one step process using *N*,*N*-dimethylacetamide (DMA) and lithium chloride (LiCl) as a solvent. CrCl_2_ with HCl and [EMIM]Cl were used as catalyst and additive, respectively. The biomass was used without any previous pretreatment. The authors demonstrated that other biomass components (lignin and protein) did not interfere in the process, as yields of HMF based on the cellulose content of the biomass were comparable to those from purified cellulose. The authors propose that the formation of HMF from cellulose in DMA-LiCl occurs via saccharification followed by isomerization of the glucose monomers into fructose and dehydration of fructose to form HMF. The saccharification of cellulose in water is thought to occur via Brønsted acid-catalyzed hydrolysis of its glycosidic bonds. A similar Lewis acid-catalyzed process could be responsible for the hydrolysis activity of chromium halides. The improved HMF yield with addition of HCl suggests that Brønsted acid catalysis also occurs in DMA-LiCl. At 140 °C and 2 h of reaction, HMF yields of 48% and 19% can be obtained from corn stover and pine sawdust respectively (Figure 7).

Another work reports the synthesis of HMF by microwave heating of sugarcane bagasse, which is an attractive raw material resource due to its composition and abundance [39]. Among different metal chloride catalysts tested, Zr(O)Cl_2_/CrCl_3_ was found to be most effective for HMF in DMA–LiCl solvent using 1-butyl-3-methyl imidazolium chloride ([BMIM]Cl) additive. An enhanced HMF yield in the presence of [BMIM]Cl was ascribed to the increase in Cl^−^ ions, which favor cellulose hydrogen bond disruption. The maximum HMF yields was 42% and the catalyst was demonstrated to be reusable.

Different parts (tuber, shoot, fruit, root or whole part) of different weed plants (red nut sedge Indian doab, marijuana, water spinach, water hyacinth, datura, yellow dock, dodder, gajar ghas, spiny pigweed, foxtail straw, wild elephant foot yam, cycus) were also used for the one pot conversion to HMF [41]. Grasses have high carbohydrate contents and can be converted into added value products by proper acid catalysts. The transformation of weed biomass to HMF includes the hydrolysis of polysaccharides to monosaccharides, isomerization of glucopyranose to fructofuranose, and dehydration of fructofuranose to HMF. The hydrolysis is believed to occur under acidic conditions in DMA–LiCl. Moreover, since commercial 99% DMA was directly used, the authors ascribed to the amount of water present in the solvent, the hydrolysis of ether linkages of polysaccharide units. Additionally, DMA–LiCl solvent facilitates the dissolution of the polysaccharides by forming DMALi^+^ macrocations, resulting in a high concentration of weakly ion paired Cl^−^ and disrupting its network of intra and inter chain hydrogen bonds. The process has been performed with ILs and silica supported immobilized heteropolyacid (HPA) catalysts under microwave-assisted heating. Foxtail weed gave maximum HMF yields: 58 and 52 wt% with [DMA]^+^[CH_3_SO_3_]^−^ and [NMP]^+^[CH_3_SO_3_]^−^ catalysts, respectively. Strong Lewis acidic silica supported heteropolyacid (HPA-SiO_2_) catalyst was also effective producing a maximum 32 wt% HMF from the same weed substrate. The spent catalyst and the solvent system proved to be recyclable. The difference in effectiveness may be due to a better proton donating ability of ILs.

Different lignocellulosic biomasses, such as filter paper, reed and straw, were converted to HMF with sulfonic acid ionic liquids and metal salt co-catalysts [42], aiming at a process not involving chromium. Using MnCl_2_ and [BMIM]Cl, at 120 °C and after 1 h of reaction, yields of 40%, 33% and 29% were obtained from filter paper, reed, and straw, respectively. The promotional effect of the manganese salt may be due to the Mn^2+^ coordination, the rapid conversion of α-glucose to β-glucose and the subsequent isomerization to fructose, which improves the yield of HMF. In particular, the authors proposed that MnCl_2_ in IL-1 forms complexes of [MnCl_2_(HSO_4_)*_n_*]^n−^ and these play a role in proton transfer, facilitating the mutarotation of α-glucose.

**Figure 8:**
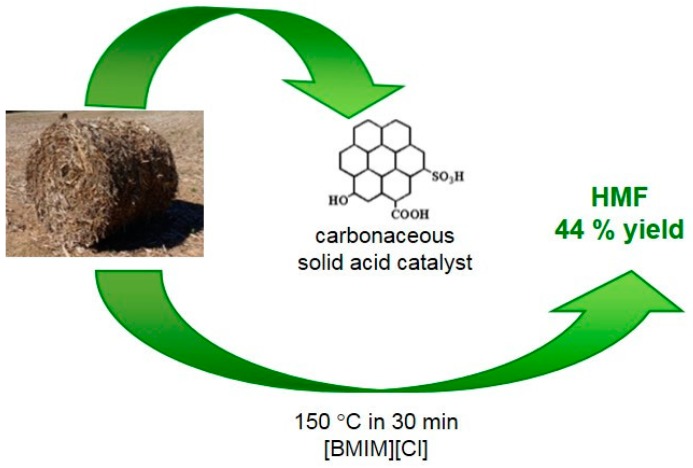
Schemes of the process from corn stalks to be used both for the synthesis of the catalyst and as raw biomass to be transformed into HMF [43].

It has been reported [43] that corn stalks can be used both for the synthesis of the catalyst and for feedstock for the one-step conversion to HMF, as schematized in Figure 8. By hydrothermal carbonization of corn stalks followed by sulfonation with concentrated H_2_SO_4_, a carbonaceous solid acid containing -SO_3_H, -COOH, -OH groups has been synthesized. The catalyst was used for HMF production in ([BMIM][Cl]). A HMF yield of 44% was achieved at 150 °C in 30 min. The authors affirmed that the good catalytic activity can be due to -OH or -COOH groups, which adsorb the cellulose molecules and then the grafted -SO_3_H groups can hydrolyze the cellulose into glucose. In addition, the -OH groups could favor the isomerization of glucose to fructose, and the fructose could be further converted into HMF catalyzed by the -COOH and -SO_3_H groups. A possible mechanism for this process was proposed. At first, biochar absorbs the corn stalk powder into its porous structure. The distributed Mg^2+^ in the catalytic system reacts with isopropanol to form active hydrogen. Meanwhile, the O-H bond of the isopropanol molecule on the catalytic surface is broken by the metal ion to form isopropyl radicals. Subsequently, the active hydrogen attacks the C-O or C-C bonds of the corn stalk segments, which causes corn stalk degradation and the formation of benzodiazepine molecules. The authors concluded that the higher conversion rate of corn stalk in this reaction system is due to the synergistic effect of the isopropanol/AMIMCl solvent and the Biochar-Mg-Sn catalyst.

The relevant importance of pretreatment conditions has been demonstrated by Wu et al. [44], who investigated for wood chips and rice straw different acid/base pretreatments (H_2_SO_4_, HCl, NaOH with different concentrations). After optimization of all parameters they observed a HMF yield around 79 mol% by pretreatment in diluted NaOH (3 wt%) solution at 60 °C for 24 h and catalysis by CrCl_3_·6H_2_O in [BMIM]Cl at 120 °C for 2 h. 

A biphasic system with IL and a magnetic metalloporphyrin catalyst was recently designed by Jiang et al. for the direct transformation of corncobs to HMF [45]. In particular, they designed a magnetic metalloporphyrin that was wrapped with chitosan in order to improve its stability. The authors affirmed that the central metal has an important role in the process because of the interaction metal-oxygen. Therefore, the activity and electric charge number of the central metal are beneficial for the activation of molecular oxygen and increase catalytic activity. They set up a process for the hydrolysis of cellulose from corncobs, recovery of glucose in the aqueous phase and finally separation of HMF. The biomass pretreatment is however a little complex: corncobs were dried, treated in water and phosphoric acid for 24 h at 4 °C, centrifuged, washed with water, added to aqueous sodium carbonate solution to adjust the pH value to 6.2, centrifuged again, washed and stored in the refrigerator. They found that a mixed catalyst (MCMP-Al, Cr and Mg) coupled with [MOMIM][PF6] could increase the HMF yield at 66%, with infrared radiation heating during distillation under reduced pressure for 50 min. The authors affirm that products could be recovered through the aqueous phase and both ILs and catalysts can be reused up to 40 times. 

**Figure 9:**
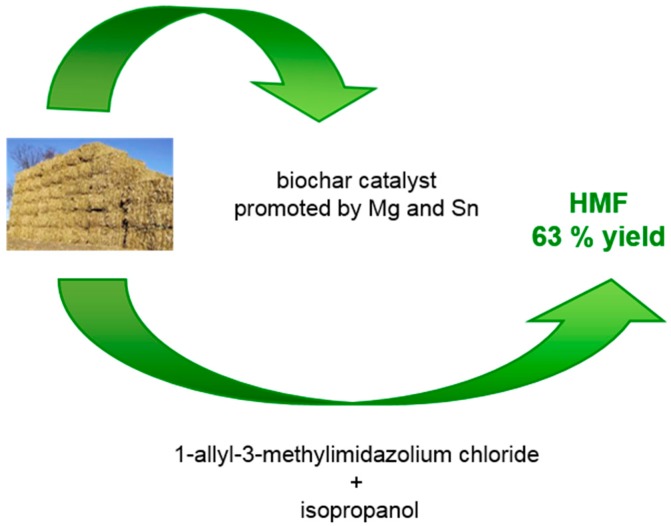
Schemes of the process from corn stalks to be used both for the synthesis of the catalyst and as raw biomass to be transformed into HMF [46].

In 2018, a study proposed the use of corn stalks to prepare biochar as a catalyst to transform the same corn stalk to HMF [46] (Figure 9). Biochar was promoted by metal ion (Mg and Sn). An ILs-organic solvent containing 1-allyl-3-methylimidazolium chloride and isopropanol was found to be the best solvent mixture. A maximum HMF yield of 63% was obtained.

Although ILs are suitable solvents for HMF synthesis [27] due to their characteristics such as low vapor pressure, good thermal stability, tunable hydrophobicity/hydrophilicity [4,28], there are too many limits to their industrial application. In particular, despite the inherent advantages of ILs, there are some limitations associated with their use, such as environment protection implications, cost of production, and their recovery. Moreover, high concentrations of HMF are a catalyst inhibitor and it is difficult to isolate HMF from ILs [25]. 

### 3.2. Other Solvents

As regard as other options for the process, Japanese red pine wood was transformed into HMF under subcritical water conditions (<374 °C and <22.1 MPa) [47]. No pretreatment of wastes (such as drying and pulverizing) is required. In fact, the authors demonstrated that there was no difference between the chip size and the yield for reactions. By using phosphate buffer at pH 2, HMF yields of about 25 wt% were obtained, but significant amounts of tar and char were formed from the pine wood.

Cassava wastes, rich in starch and cellulose, are one of the most abundant agriculture industrial wastes in Thailand [48]. In 2011, some authors proposed using them for the production of HMF using a heterogeneous catalyst based on sulfonated carbon. The best solvent for the production of HMF was found to be a mixture of acetone/dimethylsulfoxide (DMSO) (70/30% *w*/*w*) and water at a ratio of 10/90% *w*/*w*. The reaction temperature and time were 250 °C and 1 min, respectively. Under these conditions, a 12% HMF yield was obtained. The carbon-based catalyst plays an important role in enhancing hydrolysis of cellulose and hemicelluloses and promoting the dehydration of xylose and glucose to form HMF. Moreover, the sulfonated carbon-based catalyst played a role in the suppression of the glucose isomerization, while the glucose dehydration was suppressed by the presence of acetone/DMSO. Isomerization from glucose to fructose was not affected by the use of this catalyst. Moreover, the authors demonstrated the stability of such a catalytic system [48].

Another real feedstock used for HMF synthesis is bleached birch (*Betula*) kraft pulp containing both cellulose and hemicelluloses and obtained from a Finnish pulping mill [49]. The authors investigated proton forms and Pt modifications of different materials (MCM-22, MCM-48, MCM-41), Al/SBA-15, Pt/Al_2_O_3_. The authors pointed out the connection between pH and product distribution: a low pH indicates formation of sugar dehydration products, whereas formation of sugar alcohols does not give a severe drop in pH. After 24 h of reaction in water at 185 °C HMF yields are however not satisfactory (max 8%).

The transformation of sugarcane bagasse into HMF using hot compressed water (HCW) treatment was also reported [50]. The HCW treatment is a promising technique for the hydrolysis because wet biomass can be directly applied. These experiments were performed in a batch-type reactor, investigating the effects of temperature (200–300 °C) and reaction times (3–30 min). The highest yield of HMF was found at 270 °C and 10 min, but it is very low (3 wt%). At higher temperature (>300 °C) and longer reaction time, HMF yield decreased, mainly for the polymerization to formic acid and char.

A single step batch system based on the simultaneous organic solvent extraction was investigated for the transformation of maple wood to HMF [51]. Raw maple wood is considered as an example of highly recalcitrant lignocellulosic biomass. Solvent extraction could reduce xylan degradation, lower chars, and improved xylan mass balance. In fact, simultaneous extraction into a solvent immiscible in water can enhance HMF yields by removing it from the aqueous environment where the yield would otherwise be limited by its rapid degradation to chars and humins. The effectiveness of the organic solvent methyl isobutyl ketone in improving HMF yields from maple wood was demonstrated for reactions in 0.1 M sulfuric or hydrochloric acid at 170 °C. Yields of 47% HMF can be obtained.

The same authors used again maple wood biomass [52] for HMF production. They investigated the use of tetrahydrofuran (THF) as a co-solvent to enhance HMF yield. THF is relatively non-toxic, miscible with water over a wide range of reaction conditions, low boiling (66 °C), and it forms an azeotrope with water. The authors reported a one-pot monophasic reaction for the hydrolysis of maple wood to sugars, sugar dehydration, and lignin extraction. Consecutive batch reactions at 170 °C were performed using dilute sulfuric acid (1 wt%). 21% HMF yield was obtained in the liquid phase and over 90% extraction of lignin as a solid powder. 

Different lignocellulosic biomass (corn stover, pine wood, grass, and poplar) were investigated with an AlCl_3_·6H_2_O catalyst in a H_2_O/THF biphasic mixture under microwave heating [53]. After 30 min of reaction at 180 °C, HMF yields from corn stover, pine wood, grass and poplar were 19%, 35%, 23% and 26%, respectively. The addition of NaCl increases the partitioning coefficient of HMF into a biphasic system and retards the route to lactic acid, enhancing the yield and selectivity for HMF. 

The microwave process was used also for the conversion of wheat straw, which is one of the major agricultural residues in terms of costs and availability. In fact, if one considers the annual production of wheat in the world and the yield of straw (1.5 straw/grain, *w*/*w*), a large amount of wheat straw is produced as a by-product every year [54]. The process depends on temperature, time and pH, whereas liquid to solid ratio is not important. The maximum predicted HMF yields were however very low (3.4%) [55].

It has been reported that maple wood and corn stover [56] can be directly transformed into HMF with maxima yields of 51% and 45%, respectively. The authors demonstrated that metal halides are catalysts suitable for HMF production directly from lignocellulosic biomass without a separate pretreatment step. Screening of several promising metal halides AlCl_3_·6H_2_O, CuCl_2_·2H_2_O, CrCl_3_·6H_2_O, FeCl_3_·6H_2_O, and ZrOCl_2_·8H_2_O showed best results with a 1 wt% FeCl_3_ in 4:1 THF: water co-solvent system after 60 min at 170 °C. Aldose-to-ketose isomerization occurred at a faster rate than sugar dehydration. THF appeared to co-catalyze sugar dehydration by promoting a more kinetically favorable open chain dehydration way. FeCl_3_ performed best owing to its high Brønsted acidity and moderate sugar conversion rate. HMF can be concentrated by an immiscible extracting solvent and the catalyst can be recycled in the aqueous stream [56].

An interesting process has been reported in *Science* by Dumesic et al. [57]. The authors investigated the production of carbohydrates from corn stover, hardwood, and softwood in a flow-through reactor using a progressive temperature increase from 157 to 217 °C. They obtained high HMF yields (60%) in a solvent mixture of biomass-derived γ-valerolactone (GVL), water, and dilute acid (0.05 wt% H_2_SO_4_). The role of GVL is: (i) to promote thermocatalytic saccharification through complete solubilization of the biomass, including the lignin fraction; (ii) to prevents reprecipitation of lignin by-products on the surface of cellulose, which is a known phenomenon in water that decreases accessibility to the reactive cellulose surface; (iii) to play a role in disrupting cellulose crystallinity. The carbohydrates can be recovered and concentrated by addition of NaCl or liquid CO_2_.

The same corn stalks can be converted to HMF [58] by a strong acid catalyst prepared by the copolymerization of *p*-toluenesulfonic acid and paraformaldehyde. A HMF yield of 19.5% was obtained from raw corn stalk at 190 °C for 100 min in GVL.

Sugarcane bagasse, collected from a local Indian market, was also transformed into HMF after crushing into powder, and drying at 100 °C [59]. A solid acid with high surface area (1437 m^2^/g) was used as catalyst. Namely, the material was a nanoporous polytriphenylamine synthesized one-step oxidative polymerization and then sulfonated. 18.8% HMF yield was achieved in DMSO after 60 min microwave irradiation at 140 °C. 

Levulinic acid (LA) was used as a catalyst for the conversion of pinewood and eucalyptus sawdust to HMF in 2-methyltetrahydrofuran (MTHF)/water biphasic systems [60]. Eucalyptus hemicellulose contains glucuronoxylan, xylan and acetyl groups, whereas pinewood hemicellulose contains galactoglucomannans, mannose and galactose units. LA is miscible in both water and MTHF solvents, therefore, it was equally partitioned into the two phases; the presence of LA in the aqueous phase maintained the equilibrium towards HMF by preventing rehydration of HMF into LA. The use of LA as a catalyst in a biphasic system is advantageous because of: (i) better control of the side reactions; (ii) easier extraction of desirable compounds in the organic phase; (iii) higher feasibility of the product mixture to be upgraded into chemicals and fuels; (iv) absence of inorganic salts and acids. Highest yields of HMF were achieved with 1:1 *w*/*w* ratio of MTHF/water, at 180 °C and 2 h of reaction. Pinewood sawdust was found to higher yields than eucalyptus sawdust; however, both molar yields were very low (below 10%).

Other lignocellulosic biomass residues such as straw and barley husk can be transformed into HMF in one-pot, using 48 mol% of sulphanilic acid as catalyst [61]. The process worked in a biphasic solvent mixture (water/2-butanol). This is an interesting investigation due to the use of water as the main component of the solvent system, and because a pretreatment of the biomass or separation step are not necessary. Both straw and barley husk, without any pretreatment, gave 41% HMF yield. The authors affirmed that sulphanilic acid is able to simultaneously depolymerize the cellulose structure through 1,4-glycosidic bond hydrolysis and two-step isomerization and dehydration reactions to form HMF.

In 2016, Zhang et al. [62] prepared a series of heteropolyacid catalysts Ch*_x_*H_3−*x*_PW_12_O_40_ (*x* = 1, 2 and 3) with choline chloride and H_3_PW_12_O_4_. The catalysts were used in one-pot conversion of raw biomass to HMF in double solvent system containing water and methylisobutylketone. The hydrophilic head of the catalyst concentrates cellulose for catalytic conversion while the hydrophobic tail inhibits further hydration of HMF to levulinic acid. HMF yields of 27%, 11%, and 13% were achieved from corn stover, pinewood and husk of xanthoceras, at 140 °C and 10 h of reaction. The catalysts can be recycled.

In 2017 corncobs were processed into HMF via a porous polytriphenylamine–SO_3_H solid acid catalyst in lactone solvents [63]. Hydrophilic solvents demonstrated better performances than hydrophobic ones. In particular, best catalytic ability was found using GVL. In fact, the good solubility of corncob in GVL facilitates the reactions. Such GVL properties and the strong surface acidity of the catalyst are responsible for the enhanced performance. Under the optimum reaction conditions, a HMF yield of 32% was achieved at 175 °C. 

Very recently, corn stalks at five different growth stages were investigated for direct HMF production by fast pyrolysis via pyrolysis-gas chromatography/mass spectrometry technique [64]. Corn stalks include trefoil stage (30 days), elongation stage (70 days), heading stage (80 days), ripening stage (100 days) and full ripening stage (120 days). Moreover, three fractions were separated, that is leaf, stem bark and stalk pulp. The results indicated that the pyrolytic characteristics differed greatly from each other. However, results for selective production of HMF were not satisfactory. Best yield was obtained with stalk pulp at ripening stage, but it is only 5 wt% at 300 °C [64].

## 4. HMF from Food Waste

The use of FW is part of the so-called 2nd generation biorefineries, which are mainly based on non-edible biomass. In contrast to lignocellulose biorefineries, which require strict thermomechanical and chemical pretreatments, FW biorefineries utilize a biomass rich in starch and protein. In fact, even if the FW composition varies a lot from a country to another, in general, FW includes a high amount of starchy carbohydrates, fats and oils, proteins, cellulose, free soluble sugars, vitamins and minerals. These are much more suitable for acid and/or enzymatic hydrolysis to glucose, peptides and amino acids. At the same time, catalytic treatment of FW can produce different chemicals, such as monosaccharides, furans and carboxylic acids, even if humins are produced. All the investigations about HMF production from FW are very recent, as summarized in Table 3. 

Parshetti et al. in 2015 reported a thermochemical conversion in the presence of a heterogeneous catalyst (ZrP). Although the yield from FW to HMF was only 4.7%, some preliminary calculations suggested the economic feasibility of the process [65].

Yu et al., in 2016, studied the effect of SnCl_4_ catalyst in the successive conversion pathways of FW to glucose (44–65% yield) and HMF (8–9.5% yield). There was a synergistic effect of Brønsted and Lewis acid centers that allowed the hydrolysis of glycosidic bonds, fructose dehydration and glucose isomerization, as well as dehydration to HMF and the undesired polymerization to humins [66]. Other authors investigated a solid Brønsted acid (Amberlyst 36, a resin containing sulphonic groups) for the treatment of cellulosic FW; it was found that DMSO enhanced cellulose dissolution and HMF formation up to 16% in 5 min at 120 °C with respect to other solvents [67].

Also starchy FW, such as cooked rice or penne, and fruit residues were used for a one-pot process for HMF production using microwave heating at 140 °C. Yields of 23% and 13% HMF were obtained, respectively, from starchy FW and fruit residues [68]. Then the same authors investigated the effects of several aprotic solvents on the process of bread residues. Bread waste is one of the most common FW across the world and it is rich in starch that has a proven applicability for HMF production: starch is the most abundant renewable polysaccharide, it is simple, cheap and it is very interesting to investigate HMF production from starch-rich biomass. Collected samples of bread from the Hong Kong International Airport were subject to freeze-drying, grinding, and sieving. Prepared samples were then stored in an air tight storage container at 4 °C in the dark. The authors found that mixtures of water/ACN, acetone or DMSO allowed for a higher selectivity to HMF than THF/water mixtures [69]. Best results with water/ACN, while they observed that acetone/water promoted the formation of levulinic acid (17%). Other authors found a HMF yield from bread wastes of 30 mol% with a SnCl_4_ catalyst [70]. They investigated the kinetic balance between these acidities to promote desirable reactions and lower rehydration and polymerization. Preliminary economic analysis indicated a net gain for the process [70].

In 2018 the same authors used a sulfonated biochar derived from forestry wood waste for the HMF production from bread [71]. Under the optimum reaction conditions, the HMF yield of 30 mol% was achieved in the mixture of DMSO/water at 180 °C in 20 min. The effectiveness of sulfonated biochar catalyst was correlated to the density of strong/weak Brønsted acidity (-SO_3_H, -COOH, and -OH groups). The recyclability of biochar catalyst was increased by regeneration. 

Very recently, in 2018, it has been demonstrated that also beverage wastes, which represent a significant category of industrial FW [73], can be converted into HMF [72]. This waste stream is rich in sugars such as glucose, fructose, and sucrose, which are simple sugars and therefore are appealing for conversion processes. The purity and simple structure of the sugar syrup make it more feasible for chemical transformation than lignocellulosic biomass. The work [72] proposed the integration of biological and chemical processes for HMF production from mixed food and beverage wastes. In particular, the results showed that with a solid-to-liquid ratio of 70% it is possible to hydrolyze biomass by glucoamylase and sucrase to obtain a hydrolysate of glucose and fructose. After colorants and impurities removal by chromatography columns, the purified hydrolysate was processed by glucose isomerase to produce a syrup with a fructose-to-glucose ratio 1:1. By a commercial solid acid catalyst (Amberlyst 36), 71 mol% HMF was produced under microwave at 140 °C in 40 min. The authors demonstrated the reusability of the catalyst [72]. Therefore, as regard as FW transformation into HMF, the obtained yields are still not satisfactory but it has to be taken into account that researches are at the beginning.

## 5. Concluding Remarks 

To summarize, a large variety of raw feedstocks and many reaction systems have been investigated at a lab-scale level. However, conversion of biomass to HMF needs improvement in yield by developing a synergic solvent & catalyst & technological system.

First, a process that requires minimal pre-treatment of raw biomass is highly desirable and this point is still one of the main challenges for biomass conversion into HMF. In general, it is more common to pre-treat the biomass to make it more suitable for hydrolysis by increasing cellulose accessibility via removal of the lignin and hemicellulose [74] while the use of untreated raw biomass for HMF synthesis is limited. Effective strategies are needed that can efficiently fractionate raw biomass and achieve high product yields directly from biomass without expensive catalysts or solvents or complex process configurations.

While water is a well-known green option, the final yields of HMF in water are not still satisfactory due to formation of humins, insoluble structure of cellulose, the instability of HMF due to decomposition to levulinic acid or formic acid, mass transfer limitation between cellulose and the heterogeneous catalyst. Therefore, the minimization of by-product formation is a challenge in aqueous medium, so although water is a preferred option, promising product yields are often obtained in ILs. However, even if the use of ILs as solvents could be one alternative way to promote HMF production, their cost can be prohibitive and they appear to us unsuitable for a large-scale commercialization.

Because of the recalcitrance of cellulose to deconstruction, high yields at mild temperatures can be obtained by using concentrated acid. Highly corrosive and strong mineral acids like HCl, H_2_SO_4_ and H_3_PO_4_ are often employed as catalyst for the HMF synthesis. However, recovery of the mineral acid is a critical point for both economics and sustainability of the process. Few examples of solid acid catalysts were investigated for the synthesis of HMF. Solid acid catalysts have significant advantages over mineral acids because they are easily separable from the reaction mixture, nontoxic, noncorrosive, recyclable, and therefore allow to solve some of the environmental problems associated with mineral acids. High surface area and the presence of micropores/mesopores are the most crucial parameters for the efficiency of these solid acid catalysts.

As regard catalytic systems, different materials with Lewis and Brønsted acidity in aqueous solution have been investigated to improve the HMF yield. Nevertheless, HMF conversion in traditional reaction systems usually needs particular reaction conditions such as, for example, high temperature. At the same time, high loading of catalysts was also necessary to improve the reaction rate. Good yields were achieved when chromium salts were used as catalyst, but they are not environmentally friendly. Moreover, for instance, metal chlorides such as AlCl_3_ may cause neurological damage, while possible oxidation of Cr^2+^ and Cr^3+^ to carcinogenic Cr^6+^ needs careful handling [74]. Therefore, the development of an efficient, environmentally friendly catalyst is a current research hotspot. The challenge of developing a simple catalytic system and a reduced reaction temperature for producing HMF from raw biomass still remains.

Moreover, not only catalyst efficiency but also its stability and recyclability should be carefully taken into account. In fact, the complex composition of raw biomass and the presence of impurities require particular attention, yet this challenge is often overlooked in most of the investigations.

Finally, the technological barriers and engineering concerns of using real raw biomass over the heterogeneous catalysis deserve closer investigation, which determine the success in industrial applications. Thus far, relatively low feedstock loadings have been adopted in most of the lab-scale studies, which presents a hurdle to upscaling and commercialization of the conversion technology. For pilot scale production to develop into commercial scale manufacturing, an improvement in the techno-economics is missing. One possible avenue for this is by increasing solvent and catalyst recycling, which will also reduce the ecological footprint of the process. Heterogeneous catalysts have an advantage over homogeneous catalysts in this respect, but as already discussed, their cost, durability and lifespan needs to be improved. The HMF must be of sufficient purity for use as a chemical intermediate, which necessitates the complete separation and proper disposal of the by-products.

Future research on biomass valorization by heterogeneous catalysts should address all the above issues for the goal of upscaling the process for industrial applications with both a sustainable and economic point of view. In conclusion, HMF is a promising platform chemical for the future bio-based industry, but for its successfully commercialization, it is to be synthesized economically and with a minimal environmental footprint. The challenges for modern scientists remain open. 

## Figures and Tables

**Table 1:** HMF production from edible biomass.

Biomass	Liquid Ionic	Homogeneous Catalyst	Heterogeneous Catalyst	Year	Ref	HMF Yield
Cane juice		x		1948	[31]	11%
Cane juice		x	x	1969	[32]	44%
Jerusalem artichoke			x	1983	[33]	57%
Jerusalem artichoke			x	2014	[34]	35%
Chicory roots		x		1988	[35]	9%
Chicory roots	x	x		2011	[36]	51%
Grape berries	x	x		2011	[37]	10%
Tapioca roots	x	x		2012	[38]	53%

**Table 2:** HMF production from non-edible lignocellulosic biomasses.

Biomass	LIs	Homogeneous Catalyst	Heterogeneous Catalyst	Other	Year	Ref	HMF Yield
Corn stover	x	x			2009	[19]	48%
Pine sawdust	x	x			2009	[19]	19%
Sugarcane bagasse	x	x	x	mw	2012	[39]	42%
Foxtail weed	x		x	mw	2012	[41]	58%
Filter paper	x	x			2013	[42]	40%
Corn stalks	x		x		2014	[43]	44%
Wood chop rise straw	x	x			2016	[44]	79 mol%
Corncob	x		x		2017	[45]	66%
Corn stalk	x		x		2018	[46]	63%
Red pine wood		x		scwa	2010	[47]	25%
Cassava wastes			x		2011	[48]	12%
Birch kraft pulp			x		2012	[49]	8%
Corn stover		x		mw	2012	[50]	19%
Pine wood		x			2012	[53]	35%
Grass		x			2012	[53]	23%
Poplar		x			2012	[53]	26%
Wheat straw		x		mw	2012	[55]	3.4%
Sugarcane bagasse				hcw	2013	[50]	3%
Maple wood		x			2013	[51]	47%
Maple wood		x			2013	[52]	21%
Maple wood		x			2014	[56]	51%
Corn stover		x			2014	[56]	45%
Corn stover		x			2014	[57]	60%
Corn stover		x			2015	[58]	19.5%
Sugarcane bagasse			x	mw	2015	[59]	20%
Pinewood sawdust		x			2016	[60]	<10%
Straw and barley husk		x			2016	[61]	41%
Corn stover		x			2016	[62]	27%
Corncob		x			2017	[63]	32%
Corn stalks				py	2018	[64]	5%

mw = microwaves; scwa = subcritical water; hcw = hot compressed water; py = pyrolysis.

**Table 3:** HMF production from food wastes.

Biomass	Liquid Ionic	Homogeneous Catalyst	Heterogeneous Catalyst	Year	Ref	HMF Yield
Food waste			x	2015	[65]	4.7%
Food waste		x		2016	[66]	9.5%
Food waste			x	2017	[67]	16%
Cooked rise or penne waste		x		2017	[68]	23%
Fruits waste		x		2017	[68]	13%
Bread waste		x		2017	[69]	27 mol%
Bread waste		x		2017	[70]	30 mol%
Bread waste			x	2017	[71]	30 mol%
Beverage + Food wastes			x	2018	[72]	71 mol%
